# *Lindera obtusiloba* Blume Alleviates Non-Alcoholic Fatty Liver Disease Promoted by N^ε^-(carboxymethyl)lysine

**DOI:** 10.3390/nu16142330

**Published:** 2024-07-19

**Authors:** Jin-Ah Lee, Min Ji Gu, Yu Ra Lee, Yoonsook Kim, Inwook Choi, Donghwan Kim, Sang Keun Ha

**Affiliations:** 1 Korea Food Research Institute, 245, Nongsaengmyeong-ro, Iseo-myeon, Wanju-gun 55365, Republic of Korea; jalee@kfri.re.kr (J.-A.L.); gmj9656@kfri.re.kr (M.J.G.); lyr@kfri.re.kr (Y.R.L.); kimyus@kfri.re.kr (Y.K.); choiw@kfri.re.kr (I.C.); 2 Division of Food Biotechnology, University of Science and Technology, Daejeon 34113, Republic of Korea

**Keywords:** *Lindera obtusiloba* blume, advanced glycation end products (AGEs), receptor of advanced glycation end product (RAGE), non-alcoholic fatty liver disease (NAFLD), lipid metabolism

## Abstract

Non-alcoholic fatty liver disease (NAFLD) is a major issue because it is closely associated with metabolic diseases. Advanced glycation end products (AGEs) are implicated as risk factors for steatosis during NAFLD progression. AGEs influence NAFLD progression through a receptor-independent pathway involving AGE cross-link formation and a receptor-dependent pathway that binds to receptors like receptors for advanced glycation end products (RAGE). The objectives of this study are to examine the effect of *Lindera obtusiloba* Blume (LO) on NAFLD promoted by Nε-(carboxymethyl)lysine (CML), one of the most common dietary AGEs. The anti-glycation effects of LO were evaluated by inhibiting the AGEs formation and AGEs-collagen cross-links breaking. The efficacy of LO against NAFLD promoted by CML was assessed using both in vitro and in vivo models. NAFLD was induced in mice by feeding a high-fat diet and orally administering CML over a period of 12 weeks, and the effects of LO on lipid metabolism and its regulatory mechanisms were investigated. LO showed the effect of inhibited AGEs formation and breakage, and collagen cross-linking. Fed a high-fat diet with administered CML by gavage, LO administration resulted in a reduction in body weight, fat mass, serum triglycerides, total cholesterol, and low-density lipoprotein cholesterol levels. LO reduced hepatic CML accumulation and RAGE expression in mice fed a high-fat diet and orally administered CML. LO alleviated hepatic steatosis accompanied by lipid accumulation and histological damage by suppressing the expression of sterol regulatory element-binding protein 1c, carbohydrate response element binding protein, fatty acid synthase, stearoyl-CoA desaturase1, tumor necrosis factor-α, and interleukin-1β. LO alleviated the MAPK/NF-κB expression by attenuating CML and RAGE expression. Taken together, our results demonstrate that LO alleviates the progression of NAFLD by lowering the levels of AGEs by downregulating CML/RAGE expression.

## 1. Introduction

Nonalcoholic fatty liver disease (NAFLD) is associated with metabolic syndrome and most metabolic syndrome patients have NAFLD [1]. NAFLD includes simple fatty liver to steatohepatitis, a condition accompanied by hepatocyte necrosis, an inflammatory response, degenerative fibrosis, and irreversible cirrhosis. There appears to be a correlation between high-energy intake and the development of NAFLD, and several dietary components appear to influence the development of these diseases [2]. Recent studies have highlighted that excessive intake of sugars and saturated fatty acids is a significant contributor to NAFLD development. Excessive consumption of sugars, particularly fructose, has been strongly associated with increased intrahepatic triglyceride accumulation, promoting NAFLD through pathways that lead to insulin resistance and hepatic lipogenesis [3,4].

Advanced glycation products (AGEs) are generated by the Maillard reaction, which is responsible for the browning phenomenon and sweet flavor of foods observed in grilled meat, bread, and cookies, and are absorbed through food or produced naturally in the body [5]. AGEs formed due to excessive glycation reactions become resistant to decomposition and accumulate in the blood or tissues, resulting in inflammatory reactions and chronic diseases and cancer [6,7,8,9]. Therefore, many studies have been conducted to improve disease using materials with AGE-inhibitory activity. Accordingly, research and development are being conducted on materials that can inhibit AGEs or break down already formed AGE cross-links and inhibitors, including aminoguanidine (AG) and pyridoxamine (PM) [10,11]. However, most conventional AGE inhibitors are synthetic compounds that cause side effects, raising safety issues for their long-term use [12]. To date, naturally occurring antioxidants have been reported to inhibit AGE formation. However, conclusive evidence regarding their efficacy has not been provided by many clinical trials [13]. Therefore, there is a need to develop materials based on natural products that do not cause these side effects.

Recent studies have highlighted that advanced glycation products (AGEs)/receptors for advanced glycation end products (RAGE) pathway play an important role in the development and progression of NAFLD. AGEs formed through reactions between reducing sugars and proteins or lipids activate RAGE, contributing to intrahepatic lipid accumulation and promoting steatosis and non-alcoholic steatohepatitis (NASH) [14,15,16]. Moreover, inhibition of RAGE prevents NAFLD progression by blocking the nuclear factor kappa B (NF-κB) [17]. AGEs can directly influence cellular function by binding to RAGE. RAGE is integral to important physiological functions including inflammation, maintaining cellular homeostasis, and facilitating post-injury repair and regeneration via mitogen-activated protein kinases (MAPKs) and NF-κB [18]. The interaction between AGEs and RAGE triggers MAPKs and NF-κB [19]. AGE accumulation activates these signaling pathways, leading to an increased release of inflammatory factors. This induces RAGE-dependent inflammation and oxidative stress and exacerbates liver damage [20]. 

*Lindera obtusiloba* Blume (LO) is widely distributed across East Asia, including in Korea, Japan, and China, and is considered traditional medicine for inflammatory diseases. It has long been used in oriental medicine to treat chronic liver disease [21]. LO has been reported to have antidiabetic [22], anti-obesity [23], hepatoprotective [24], and anti-hyperlipidemic activities [25]. However, there have been no reports of its AGE inhibitory activity. Therefore, we aimed to determine the AGE-inhibitory activity and improvement of AGE-triggered NAFLD in LO.

## 2. Materials and Methods

### 2.1. Preparation of LO Extract

Dried LO leaves were purchased from Chengmyeong Herbal Medicine Co., Ltd. (Yeoju-si, Gyeonggi-do, Republic of Korea). LO was crushed and dissolved in 50% fermented alcohol (1:10, *w*/*v*) and then extracted twice through reflux cooling extraction at 50 °C for 3 h. The filtered extract by Whatman no.1 filter paper was concentrated using a rotary evaporator (Büchi Labortechnik AG, Flawil, Switzerland). Subsequently, the concentrate was freeze-dried, and extract was stored at −20 °C. 

### 2.2. Identification of LO Extract Marker Compound by High-Performance Liquid Chromatography (HPLC)

The LO extract was analyzed to determine the presence of the marker compound quercitrin, which served as the reference compound, as previously described [26]. To prepare both the sample and reference standard solutions, the material (0.5 mg) was dissolved in 1 mL of 70% methanol solution.

HPLC was performed under specific conditions using a JASCO HPLC system equipped with a Zorbax Eclipse XDB-C18 column (250 × 4.6 mm, particle size: 5 μm) and a UV–Vis detector. Chromatographic separation was performed using a gradient elution system comprising 0.5% acetic acid in water (solvent A) and methanol (solvent B). The temperature was maintained at 30 °C during analysis. The gradient elution program was as follows: 0–5 min at 38% B; 5–30 min at 38–42% B; 30–33 min at 42–45% B; 33–35 min at 45–48% B; 35–50 min at 48–50% B; 50–58 min at 50–85% B; 58–59 min at 85–38% B. Prior to initiating the analysis of the subsequent samples, the system was held at 38% solvent B for 1 min. Each analytical run had a total run time of 60 min, with an injection volume of 10 μL and a constant flow rate of 1.0 mL/min. The detection was performed using UV light at a wavelength of 342 nm. A calibration curve was constructed using a standard solution of quercitrin at concentrations ranging from 1.56 to 100 μg/g.

### 2.3. Determination of Anti-Glycation

To measure the effect of LO on inhibiting AGE formation, 10 mg/mL bovine serum albumin (BSA; Sigma-Aldrich, St. Louis, MO, USA) was dissolved in 50 mM phosphate buffer (pH 7.4) and incubated with 0.5 M fructose. Next, 0.02% sodium azide was added to prevent bacterial growth during the reaction. After adding various concentrations of LO extract or 1 mM aminoguanidine (AG, AGEs formation inhibitor), the reaction was carried out in the dark at 50 °C for 24 h. AGE formation was quantified by measuring the fluorescence intensity at an excitation and emission wavelength of 350 nm and 450 nm, respectively, using a microplate reader (Molecular Devices, Sunnyvale, CA, USA). The formation rate of AGEs (%) was calculated using the following equation: AGEs formation (%) = (fluorescence intensity in the presence of LO extract − fluorescence intensity of blank)/(fluorescence intensity in the absence of LO extract − fluorescence intensity of blank) × 100.

To confirm the ability of LO to break the AGEs and collagen cross-linking, solution containing 5 μg/mL horseradish peroxidase-labeled AGEs was dispensed into a collagen-coated 96-well plate and incubated at 37 °C for 4 h to allow the formation of cross-linked AGEs and collagen. Then 96-well plate was washed three times with 0.05% PBST (phosphate buffered saline with 0.05% Tween 20), followed by incubation with different concentrations of LO extract or 100 μg/mL of alagebrium (ALT-711, AGEs cross linkage breaker) at 37 °C for 18 h. Uncross-linked AGEs were removed by washing three times with 0.05% PBST. 3,3′,5,5′-Tetramethylbenzidine (TMB) substrate solution was added to develop the color, and 1 N HCl was added to terminate the reaction. Absorbance was measured at 450 nm using a microplate reader (Molecular Devices, Sunnyvale, CA, USA). The breaking of AGE–collagen cross-linking (%) was calculated following equation: AGE–collagen cross-linking breaking (%) = 1 − [(absorbance in the presence of LO extract)/(absorbance in the absence of LO extract)] × 100.

### 2.4. Cell Culture and Treatment

AML-12 cells were purchased from the American Type Culture Collection (ATCC, Rockville, MD, USA). The cells were cultured in DMEM/F12 (1:1) medium (Gibco, Grand Island, NY, USA) containing 1× insulin–transferrin–selenium (Gibco), 10% fetal bovine serum (Gibco, Grand Island, NY, USA), and 1% penicillin/streptomycin (Gibco). 

For measurement of intracellular CML and Oil Red O (ORO; St. Louis, MO, USA) staining, 25 and 50 μg/mL of LO extract was pretreated for 1 h, followed by treatment with 0.5 mM of free fatty acid (FFA; oleic acid and palmitic acid in a 2:1 ratio) and/or 0.5 mM of free fatty acids and CML (FC) for 24 h.

### 2.5. Assessment of Cell Viability

The cells were incubated with various concentrations (0, 25, 50, 100, and 200 μg/mL) of the LO extract for 24 h. After removing the supernatant, 0.1 mg/mL 3-(4,5-dimethylthiazol-2-yl)-2,5-diphenyltetrazolium bromide (MTT) reagent was added and the cells were incubated for 4 h. Absorbance was measured at 570 nm using a microplate reader (Molecular Devices). 

### 2.6. Measurement of Intracellular N^ε^-(carboxymethyl)lysine (CML)

The cells were homogenized using RIPA buffer (Sigma-Aldrich) then lysate was measured using the OxiSelect^TM^ CML competitive ELISA kit (Cell Biolabs Inc., San Diego, CA, USA). We used the CML conjugated with BSA (CML-BSA) standard supplied by the manufacturer. A standard curve was generated in the concentration range from 0 to 12.5 μg/mL using this standard material, and the concentration of CML was calculated accordingly. Intracellular CML levels were determined using the standard curve obtained from a reaction with a standard substance.

### 2.7. ORO Staining for Hepatocyte

For ORO staining, cells were washed with PBS, and then 10% formalin was added and fixed for 30 min. Formalin was discarded by washing it three times with distilled water (DW). ORO staining solution was added to the cells in the dark for 20 min. The ORO staining solution was discarded, and the cells were washed five times with DW. The cells were observed at a magnification of 200×. Cells were incubated in isopropanol for 15 min to dissolve the stained ORO. The density was measured at 510 nm.

### 2.8. Animals

C57BL/6J male mice (6 weeks) were purchased from the Central Laboratory Animal (Seoul, Republic of Korea). All animal experiments followed the guidelines of the Animal Care and Use Committee of the Korea Food Research Institute (KFIR-M-22019). The mice were housed under consistent conditions of 23 ± 1 °C, humidity 55 ± 5%, and a 12 h light/dark cycle. The animals were provided food and water ad libitum. 

The mice were randomly divided into the following five groups (*n* = 9 per group): (1) normal diet (ND), mice fed an ND (AIN-93G diet; Research Diet, Inc., New Brunswick, NJ, USA); (2) HFD + CML (HFCML), mice fed 45% kcal HFD (D12492; Research Diet, Inc., New Brunswick, NJ, USA) + CML 10 mg/kg/day; (3) HFCML + LO extract 100 mg/kg/day (LO-L); (4) HFCML + LO extract 200 mg/kg/day (LO-H); and (5) HFCML + PM 10 mg/kg/day (positive control, AGEs inhibitor). CML (100 μL) and LO extracts (100 μL) were administrated once daily by gavage for 12 weeks. CML, LO, and PM were dissolved in DW and orally administered at the described dose. The ND group received an equivalent volume of DW orally for 12 weeks. Body weight was measured once weekly during the 12 weeks of the experiment. After 12 weeks, body composition scans were performed using InAlyzer (Medikors Inc., Seongnam, Republic of Korea). Scanning images and body fat mass were obtained using the InAlyzer software (Medikors Inc., Seongnam, Republic of Korea, version 1.4.6). The mice were fasted for 12 h, euthanized by isoflurane exposure, and their blood was collected. Serum samples were centrifuged at 1500× *g* for 15 min.

### 2.9. Biochemical Analysis

Serums were analyzed for triglyceride (TG), total cholesterol (TC), low-density lipoprotein cholesterol (LDL-c), and high-density lipoprotein cholesterol (HDL-c) levels. TG levels were measured using a kit from Cayman (Ann Arbor, MI, USA). TC, LDL-c, and HDL-c levels were measured using a Cell Biolabs kit (Beverly, MA, USA).

### 2.10. Histology and Immunohistochemistry

Ten percent of formalin was used to fix liver tissues, embedded in paraffin, and sectioned into 4 μm thick slices. To evaluate liver injury and lipid accumulation, sections were stained with hematoxylin and eosin, and ORO. Immunohistochemistry (IHC) was performed to monitor N^ε^-(carboxymethyl)lysine (CML; KH011; Trans Genic Inc. Kobe, Japan) accumulation and measure receptor expression of advanced glycation end products (RAGE; ab3611; Abcam, Waltham, Boston, MA, USA). All images were acquired using a digital slide scanner (Motic EasyScan One, Motic, Hong Kong) at 10× magnification. 

### 2.11. Western Blotting

Liver tissues were homogenized with RIPA buffer (Sigma-Aldrich) supplemented with protease/phosphatase inhibitor (Cell Signaling Technology, Danvers, MA, USA). The protein quantification was measured using a BCA (Thermo Fisher Scientific, Waltham, MA, USA). Proteins were separated by SDS-PAGE and transferred to nitrocellulose membrane. Following primary antibodies were incubated overnight at 4 °C: SREBP-1c (sc-365513; Santa Cruz, Dallas, TX, USA), ChREBP (#58069; Cell Signaling Technology), FAS (610963; BD Biosciences, Franklin Lakes, NJ, USA), SCD1 (#2438; Cell Signaling Technology), TNF-α (#11948; Cell Signaling Technology), IL-1β (sc-52012; Santa Cruz), phosphor-JNK (#9255; Cell Signaling Technology), JNK (#9258; Cell Signaling Technology), phospho-ERK (#9101; Cell Signaling Technology), ERK (#9102; Cell Signaling Technology), phosphor-IκB (sc-8404; Santa Cruz), NF-κB (ac-8008; Santa Cruz), or β-actin (sc-74448; Santa Cruz). Primary antibodies were diluted at 1:1000 before use. Following primary antibody incubation, the blots were washed and incubated with HRP-conjugated secondary antibodies (#7074 and #7076; Cell Signaling Technology) for 1 h at room temperature. Secondary antibodies were diluted at 1:5000 before use. Proteins were detected using enhanced chemiluminescence (ECL) reagents and a ChemiDoc XRS+ imaging system (Bio-Rad Inc., Hercules, CA, USA). The expression of each protein was quantified using Image Lab software (Bio-Rad Inc., version 6.1).

### 2.12. Statistical Analysis

The data are expressed as mean ± standard error of the mean at least three independent experiments. Differences between groups were analyzed for statistical significance using one-way analysis of variance, followed by Tukey’s multiple comparison test as a post hoc test. Statistical significance was set at *p* < 0.05. Statistical analyses were performed using the SPSS software (version 20.0; IBM Corp., Armonk, NY, USA).

## 3. Results

### 3.1. HPLC Analysis of LO Extract

**Figure 1:**
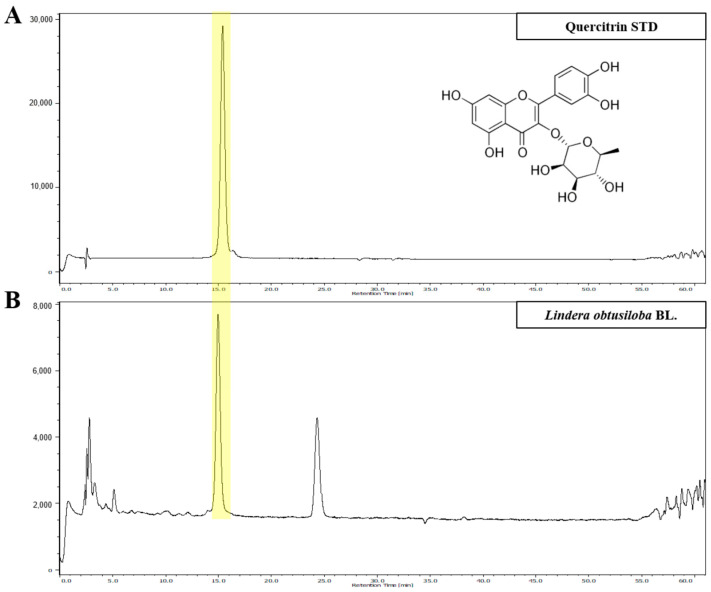
HPLC analysis of *Lindera obtusiloba* Blume (LO) ethanolic extract. The high-performance liquid chromatography (HPLC) chromatogram of standard solution (**A**) and *Lindera obtusiloba* Blume extract (**B**).

HPLC analysis of LO extract revealed the presence of quercitrin (Figure 1). Marked in yellow indicate the retention time [15.2 min] of quercitrin appearing in the standard sample (Figure 1A) and the LO extract (Figure 1B). Quercitrin concentration in the LO extract was 38.59 ± 2.71 μg/g. The HPLC method was validated by assessing various parameters, including linearity, limitation of detection, and limitation of quantification (Table 1). Collectively, these results demonstrate the suitability of HPLC for the characterization of quercitrin in the LO extract.

### 3.2. Effect of LO on Anti-Glycation

**Figure 2:**
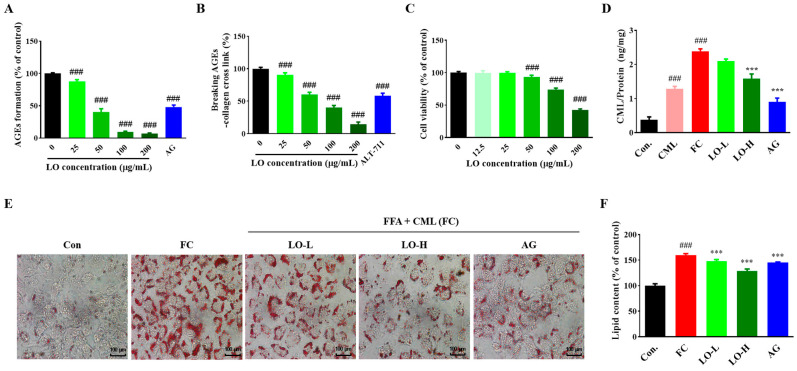
Effect of *Lindera obtusiloba* Blume (LO) on activity anti-glycation and hepatic lipid accumulation. AGEs formation suppression (**A**) and cross-linking formed breaking between AGEs and collagen (**B**) in LO. (**C**) Cell viability, (**D**) CML level, (**E**) ORO staining, and (**F**) quantification of stained ORO in free fatty acid and CML (FC)-induced AML-12 cells. All results are presented as mean ± standard error of the mean. ^###^ *p* < 0.001 versus controls; *** *p* < 0.001 versus FC. (AG: aminoguanidine, CML: N^ε^-(carboxymethyl)lysine, FFA: free fatty acid mixture (oleic acid and palmitic acid in the ratio of 2:1), FC: FFA and CML.

To explore the effect of LO on the AGEs formation inhibition, LO extract was added to a reaction that induced the formation of AGEs, using BSA as a protein source and fructose as a sugar source. The incubation of BSA with fructose substantially induced AGE formation. However, the AGEs formed by the reaction between BSA and fructose were reduced by the LO extract in a concentration-dependent manner (*p* < 0.001) (Figure 2A). Especially, 200 μg/mL of LO extract inhibited AGE formation by 93.19 ± 0.95%. This indicates a significant inhibition of AGE formation compared to the positive control AG (48.01 ± 3.08%) (*p* < 0.001). These results indicate that LO can inhibit AGE formation by inhibiting BSA glycation by fructose.

AGEs accumulate intracellularly through irreversible cross-linking with extracellular matrix proteins such as collagen. To determine the AGE–collagen cross-link-breaking ability of LO, we added AGE–BSA to a collagen-coated plate to cross-link them and evaluated the efficacy of LO in AGEs and collagen cross-link breaking. As the concentration of LO extract increased, AGE–collagen cross-link significantly decreased, and at 200 μg/mL LO extract, cross-link breaking was 85.49 ± 3.39%, showing high cross-link breaking efficacy (*p* < 0.001). In the case of ALT-711, a positive control group, the cross-link breaking was 41.64 ± 3.96% (*p* < 0.001) (Figure 2B). These findings suggest that LO inhibits the AGE formation and breaks AGE–collagen cross-linking.

### 3.3. Effect of LO on Anti-Glycation and Inhibition of Lipid Accumulation in AML-12 Cells

LO extract (0, 12.5, 50, 100, and 200 μg/mL) were treated to AML-12 cells, and cell viability was confirmed using MTT assay. Cytotoxicity was observed at LO extract concentrations above 100 μg/mL (Figure 2C). Thus, we used non-toxic concentrations of 25 and 50 μg/mL LO extract in subsequent experiments.

As shown in Figure 2D, intracellular CML levels were 1.29 ± 0.07 ng/mg in CML treatment alone and 2.39 ± 0.07 ng/mg in FC treatment. Intracellular CML levels significantly increased with FC treatment compared to CML treatment alone (*p* < 0.001). However, intracellular CML levels were decreased dose-dependently with the LO extract (2.08 ± 0.05 ng/mg and 1.45 ± 0.04 ng/mg, respectively).

Hepatic steatosis was induced by treating hepatocytes with FC and the inhibition of lipid accumulation by LO was evaluated. The lipid content was increased by FC treatment but decreased by LO treatment (*p* < 0.001) (Figure 2E,F).

### 3.4. Effect of LO on Body Weight and Serum Biochemical Markers in Mice

**Figure 3:**
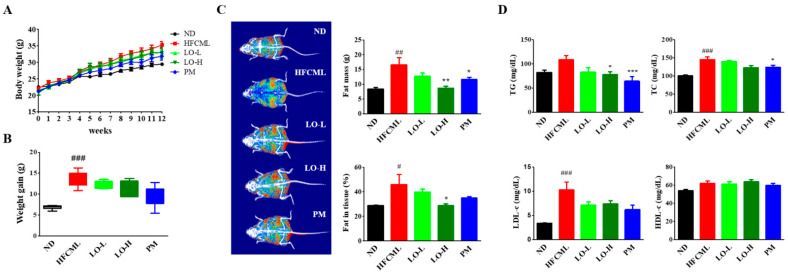
Metabolic parameters of the HFCML mice. Body weight change (**A**) and gain (**B**) during the 12 weeks. (**C**) Representative images of body composition and measurements of fat tissue mass and percentage. (**D**) Serum TG, TC, LDL-c, and HDL-c levels. All results are presented as mean ± standard error of the mean (*n* = 9). ^#^ *p* < 0.05, ^##^ *p* < 0.01 and ^###^ *p* < 0.001 versus ND; * *p* < 0.05, ** *p* < 0.01 and *** *p* < 0.001 versus HFCML. ND, normal diet; HFCML, high-fat diet and CML administration (10 mg/kg/p.o.); LO-L, HFCML + low dose of LO (100 mg/kg/p.o.); LO-H, HFCML + high dose of LO (200 mg/kg/p.o.); PM, HFCML + pyridoxamine (10 mg/kg/p.o.).

The body weight was monitored weekly over a period of 12 weeks to assess changes in body weight. The HFCML group exhibited significantly higher values compared to the ND group. Moreover, a significant increase in fat percentage was noted in the HFCML group compared to the ND group (*p* < 0.001). However, the LO group showed a reduction in body weight and fat percentage (Figure 3A–C). As expected, serum triglyceride, total cholesterol, and LDL-c levels were significantly elevated in the HFCML group than in the ND group (*p* < 0.01). However, these increases in the HFCML group decreased in the LO group (Figure 3D). High-density lipoprotein cholesterol (HDL-c) did not show any significant changes in any of the groups (Figure 3D).

### 3.5. Effect of LO on Histological Parameter in Liver Tissue

**Figure 4:**
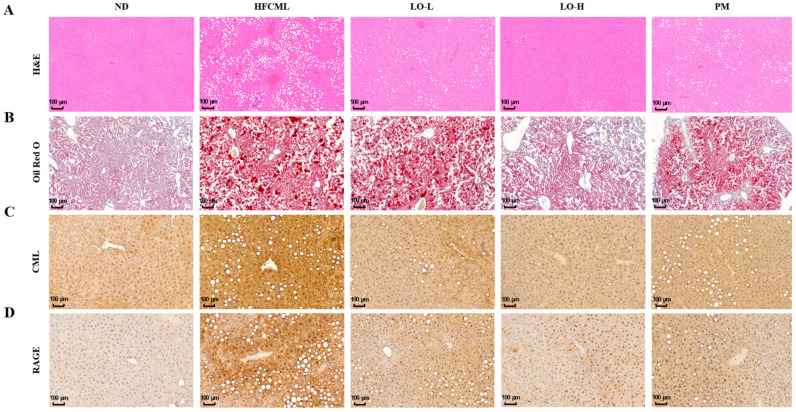
Effect of LO on histological parameter in liver tissue of HFCML mice. Representative microscopic images of liver sections stained with hematoxylin and eosin ((**A**); magnification: 10×), ORO ((**B**); magnification: 10×), CML ((**C**); magnification: 10×) and RAGE ((**D**); magnification: 10×) in liver tissues. ND, normal diet; HFCML, high-fat diet and CML administration (10 mg/kg/p.o.); LO-L, HFCML + low dose of LO (100 mg/kg/p.o.); LO-H, HFCML + high dose of LO (200 mg/kg/p.o.); PM, HFCML + pyridoxamine (10 mg/kg/p.o.).

Histological examination of liver tissues revealed excessive lipid levels in the HFCML group. In the HFCML group, steatosis and lobular inflammation were more severe than those observed in the ND group. The LO group showed markedly reduced excess lipids and inflammation compared with the HFCML group (Figure 4A,B). These changes seem to correlate with changes in body weight, body fat percentage, and biochemical markers.

CML interacts with RAGE, promoting the onset of hepatic steatosis and inflammation. To investigate the relationship between hepatic steatosis development and the accumulation of CML, we performed IHC in the liver. In the ND group, the liver showed a very weak staining for CML, whereas the liver of the HFCML group exhibited a strong in the HFCML group. In the LO group, the liver showed CML staining levels that were notably lighter, comparable to those observed in the ND group (Figure 4C). This result indicates that LO significantly reduced the CML accumulation induced by HFCML. To determine whether liver damage caused by lipid accumulation and inflammation is related to RAGE, we measured RAGE expression in liver tissue. Basal RAGE expression is usually low and elevated in pathological conditions [19]. RAGE staining was not observed in the liver tissue of the ND group; however, RAGE expression clearly increased in the liver tissue of the HFCML group (Figure 4D). Increased RAGE expression in the HFCML group was significantly decreased in the LO group. NAFLD is characterized by hepatic steatosis and damage. Our results suggest that LO alleviates NAFLD by inhibiting CML accumulation and by decreasing RAGE expression.

### 3.6. Effect of LO on Hepatic De Novo Lipogenesis (DNL)

**Figure 5:**
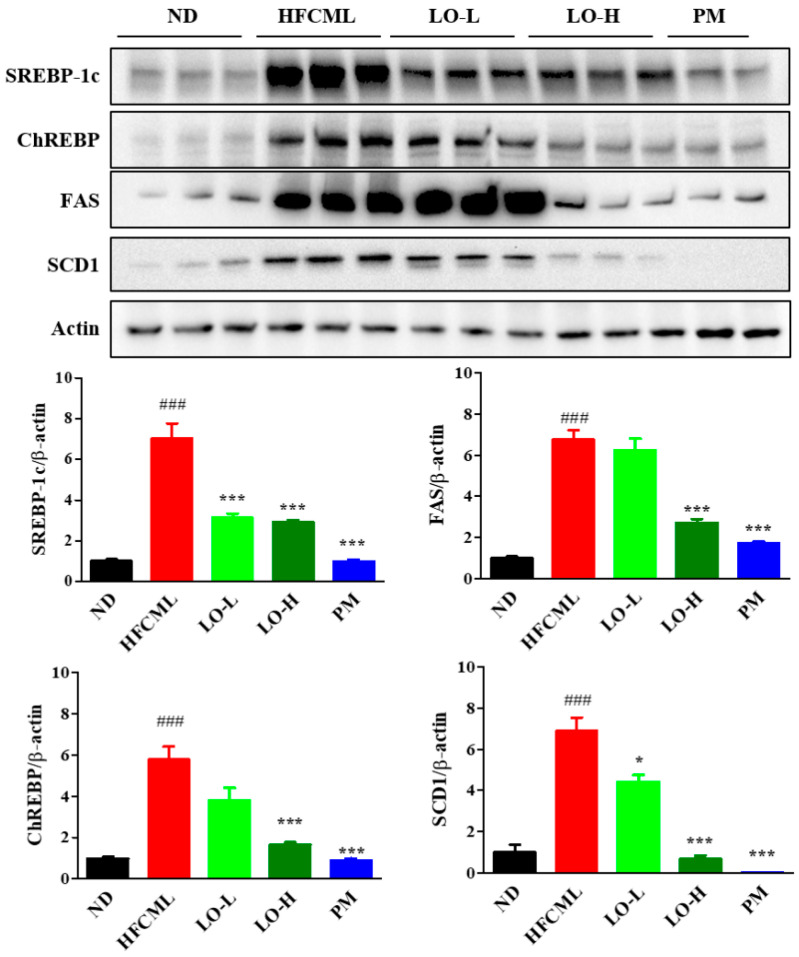
Effect of LO on lipid metabolism in liver tissues of HFCML mice. Western blot and quantification analysis of SREBP-1c, ChREBP, FAS, and SCD-1 proteins in liver tissue. All results were normalized to the ND and presented as mean ± standard error of the mean (*n* = 3). ### *p* < 0.001 versus ND; * *p* < 0.05 and *** *p* < 0.001 versus HFCML. ND, normal diet; HFCML, high-fat diet and CML administration (10 mg/kg/p.o.); LO-L, HFCML + low dose of LO (100 mg/kg/p.o.); LO-H, HFCML + high dose of LO (200 mg/kg/p.o.); PM, HFCML + pyridoxamine (10 mg/kg/p.o.).

To determine whether CML accumulation in hepatic steatosis is implicated in the induction of lipid metabolism, we evaluated sterol regulatory element binding protein (SREBP)-1c, carbohydrate response element binding protein (ChREBP), fatty acid synthase (FAS), and stearoyl-CoA desaturase1 (SCD1) proteins expression in the liver. The expression levels of SREBP-1c, ChREBP, FAS, and SCD1 in the liver were significantly lower in the LO group than in the HFCML group (*p* < 0.001) (Figure 5). These data indicate that LO inhibits hepatic lipid accumulation by downregulating lipid metabolism.

### 3.7. Effect of LO on MAPK/NF-κB Regulation 

**Figure 6:**
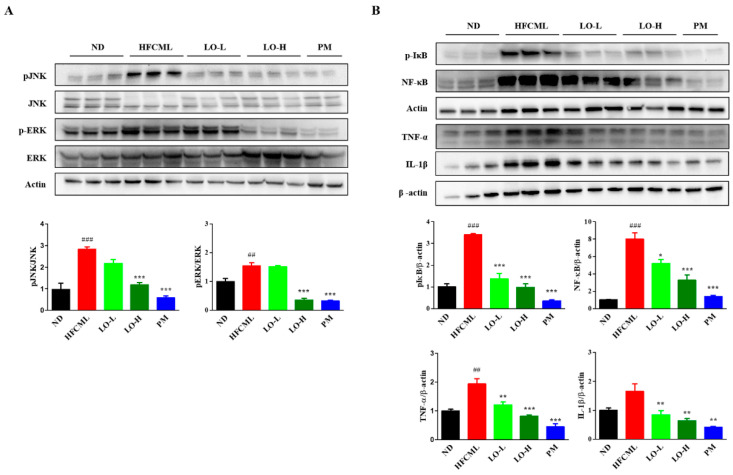
Effect of LO on modulation of MAPK/NF-κB expression in HFCML mice liver. (**A**) Western blot and quantitation analysis of *p*-JNK and *p*-ERK proteins in liver tissue. (**B**) Western blot and quantitation analysis of phosphorylation IκB, NF-κB, TNF-α and IL-1β proteins in liver tissue. All results were normalized to the ND and presented as mean ± standard error of the mean (*n* = 3). ## *p* < 0.01 and ### *p* < 0.001 versus ND; * *p* < 0.05, ** *p* < 0.01 and *** *p* < 0.001 versus HFCML. ND, normal diet; HFCML, high-fat diet and CML administration (10 mg/kg/p.o.); LO-L, HFCML + low dose of LO (100 mg/kg/p.o.); LO-H, HFCML + high dose of LO (200 mg/kg/p.o.); PM, HFCML + pyridoxamine (10 mg/kg/p.o.).

MAPK/NF-κB pathway plays a pivotal role in NAFLD and insulin resistance. Activation of this pathway by AGEs binding to RAGE influences hepatic lipid deposition, insulin resistance, inflammation, and fibrosis [20]. As LO alleviates CML-induced NAFLD, we explored the effect of LO on the MAPK/NF-κB signaling pathway. HFCML significantly increased Jun *N*-terminal kinase (JNK) phosphorylation by 2.3-fold and extracellular signal-regulated kinase (ERK) by 1.5-fold compared to ND (Figure 6A). LO attenuated the phosphorylation of JNK and ERK compared to HFCML (*p* < 0.001). In addition, phosphorylation of IκB was increased in HFCML, whereas it was downregulated by LO (*p* < 0.001) (Figure 6B). NF-κB activation was elevated in HFCML compared to ND (*p* < 0.001). In contrast, LO was suppressed, particularly in the LO-H group (*p* < 0.001) (Figure 6B). The LO group showed a significant decrease in TNF-α and IL-1β expression compared with the HFCML group (*p* < 0.01) (Figure 6B). The LO group showed a significant decrease in the expression of TNF-α and IL-1β compared with the HFCML group (*p* < 0.01) (Figure 6B). These results suggest that LO downregulated the MAPK/NF-κB signaling pathway.

## 4. Discussion

Non-enzymatic glycation reaction generates AGEs in the body, often as a result of excessive intake of carbohydrates or fat [27]. AGEs in the body are resistant to degradation and accumulate in the tissues. Accumulated AGEs form cross-links between basement membrane molecules, which abnormally change the structure and function of the tissue. AGEs accelerate disease progression by binding to receptors and activating various cell-signaling pathways [28]. Therefore, many studies have been conducted on the disease alleviation by substances with AGE-inhibitory activity. Accumulating evidence showed that LO extracts have health-beneficial compounds that modulate oxidative stress and inflammation [29]. However, the effect of LO on glycation and AGEs-induced NAFLD is unknown. The present study is about the effect of LO extracts on AGEs-induced NAFLD.

According to the HPLC analysis, quercitrin was the main compound in the LO extract (Figure 1). Quercitrin inhibits the formation of AGEs during the glycation process [30]. Additionally, it can inhibit AGEs cross-link and break those already formed [31]. LO extract containing compounds with AGEs inhibitory activity inhibited AGEs formation and enhanced the ability to break AGEs cross-links. Based on these results, we selected CML as representative free AGEs to study the effect of LO on alleviating AGE-triggered NAFLD. CML is formed from proteins during food processing and under physiological conditions in vivo. To induce AGE-triggered NAFLD, the hepatocytes were treated with FC. LO reduced CML levels, which were increased by FC. It also inhibited FC-induced hepatic lipid accumulation. This suggests that LO inhibits hepatic lipid accumulation by inhibiting AGEs.

NAFLD is a disease in which excessive amounts of triglycerides accumulate in the liver [32]. The latter stage is characterized by inflammation and steatosis. Liver inflammation has several histological features that distinguish it from steatosis, including hepatocellular and lobular inflammation. AGEs exert their harmful effects through several receptors. RAGE is a major receptor-bound AGE [33]. The binding of AGEs and RAGE triggers multiple signaling pathways that regulate gene expression. High intake of dietary AGEs worsens liver damage and inflammation [20,34]. Similarly, dietary AGEs promoted liver fibrosis from simple steatosis [35]. Additionally, among patients with NAFLD, those with high serum AGE levels had a 4.6-fold higher risk of developing severe steatosis than those with low serum AGE levels [36]. Our recent findings showed that a diet containing AGEs may exacerbate NAFLD via lipogenic mechanisms [37]. As depicted in Figure 2, when AML-12 cells were treated with FC, the amount of CML and lipid accumulation increased compared to CML treatment alone. In contrast, LO treatment significantly reduced the CML levels and lipid accumulation. To confirm these results, the mice were fed HFCML for 12 weeks. Our results identified changes in the biomarkers of steatosis, such as an increased fat proportion and increased blood triglyceride and cholesterol levels, in the HFCML group. These changes were mitigated by treatment with LO. Histological findings in the liver indicated that the HFCML group showed increased fat accumulation and lobular inflammation, both of which were alleviated in the LO group. Additionally, the expression of CML and RAGE in the liver increased in the HFCML group but significantly decreased in the LO group. These findings indicate that CML exacerbates NAFLD, whereas LO reduces CML and RAGE expression, thereby inhibiting lipid accumulation and liver inflammation.

SREBP-1c and ChREBP are transcription factors that regulate hepatic DNL [38]. SREBP-1c and ChREBP modulate DNL and triglyceride synthesis-related gene expression including acetyl-CoA carboxylase, FAS, and SCD1 [39,40]. Their expression converts fatty acids into triglycerides, and excess triglycerides lead to lipid accumulation in the hepatocytes. We found that SREBP-1c and ChREBP expression was increased in HFCML-fed mice liver. In addition, FAS and SCD1 expressions, which are downstream targets of these two transcription factors, were significantly increased. By contrast, LO treatment suppressed the expression of these proteins. These observations suggest that LO alleviates steatosis induced by the HFCML diet by modulating DNL. 

Hepatocytes are damaged by lipids and their accumulated metabolites and promote the release of inflammatory cytokines, thereby continuing the inflammatory response. These inflammatory cytokines are closely associated with NAFLD onset and prognosis [41]. Several factors simultaneously exert synergistic effects on the progression of steatosis into steatohepatitis. AGEs are also key players in this process. The AGE formation can lead to loss of hepatocyte function and AGE accumulation in the liver promotes inflammation and fibrosis through interaction with RAGE [42]. We confirmed that CML accumulation results in lipid accumulation and inflammation in the liver. Moreover, the expression of TNF-α and IL-1β increased in the livers of the HFCML group. In contrast, LO treatment decreased the expression of these proteins. These results suggest that CML is responsible for the upregulation of inflammatory markers, whereas LO reduces CML accumulation and downregulates inflammatory markers. 

The AGE accumulation can trigger pro-inflammation and fibrotic pathways, leading to alterations in the structure and function of the extracellular matrix [43]. When pro-inflammatory cytokine production increases, RAGE is upregulated, producing more pro-inflammatory cytokines [44]. Similarly, blocking RAGE can inhibit pro-inflammatory gene expression [42]. Activated RAGE stimulates the MAPK/NF-κB pathway, which is one of the most extensive mechanisms of cell regulation. Activation of the MAPK/NF-κB pathway causes liver damage through upregulating inflammatory factors, while triglyceride accumulation in the liver serves as a protective mechanism against damage [42,45,46]. NF-κB is involved in immune and inflammatory responses. Once activated, they translocate to the nucleus via the MAPK pathway and induce inflammation-related response [47]. NF-κB activation can promote AGEs and RAGE binding and further promote the sustained expression of cytokines and tissue factors [48]. RAGE promoter region has an NF-κB binding site, therefore upregulation of RAGE itself affects NF-κB activation. AGE–RAGE interaction in the liver accelerated the pathogenesis of liver disease [35,49]. We observed a significant increase in phosphorylation of JNK and ERK in the HFCML group. Conversely, the LO group exhibited a decrease in the phosphorylated JNK and ERK protein levels. Furthermore, our results showed that the phosphorylation of IκB and NF-κB was increased in the HFCML group, while it was significantly decreased in the LO group. This indicates that LO reduces lipid metabolism- and inflammation-related proteins by suppressing the MAPK pathway and subsequently suppressing the NF-κB expression. Thus, LO alleviates AGE-induced NAFLD triggered by AGEs.

## 5. Conclusions

This study elucidates how LO attenuates AGE-induced NAFLD exacerbated by AGEs. The present study has limitations regarding the inhibitory mechanism of AGE and RAGE by LO in mice models. However, LO inhibits AGE formation and increases the breakage of AGE cross-links. Moreover, LO reduced CML and RAGE expression, thereby inhibiting the induction of the MAPK/NF-κB expression. LO decreases lipid metabolism and inflammation-related protein expressions, ultimately ameliorating hepatic steatosis. Taken together, these results suggest that LO acts as an AGE inhibitor and alleviates AGE-triggered NAFLD by downregulating the MAPK/NF-κB expression. 

## Figures and Tables

**Table 1:** Analytical results of linearity, limit of detection (LOD), and limit of quantification (LOQ) level of quercitrin.

Linear Regression Equation	Correlation Coefficient (R²)	Linear Range (μg/g)	LOD (μg/g)	LOQ (μg/g)
y = 8321x − 7022	0.9995	1.56–100.00	0.60	1.81

## Data Availability

The original contributions presented in the study are included in the article, further inquiries can be directed to the corresponding author.
